# Gold nanoparticle-polymer nanocomposites synthesized by room temperature atmospheric pressure plasma and their potential for fuel cell electrocatalytic application

**DOI:** 10.1038/srep46682

**Published:** 2017-04-24

**Authors:** Ri-Chao Zhang, Dan Sun, Ruirui Zhang, Wen-Feng Lin, Manuel Macias-Montero, Jenish Patel, Sadegh Askari, Calum McDonald, Davide Mariotti, Paul Maguire

**Affiliations:** 1 School of Materials Science and Engineering, East China Jiaotong University, Nanchang 330013, China; 2 School of Mechanical and Aerospace Engineering, Queen’s University, Belfast, BT9 5AH, UK; 3 Nanotechnology and Integrated Bioengineering Centre (NIBEC), Ulster University, Northern Ireland, BT37 0QB, UK; 4 Department of Chemical Engineering, Loughborough University, Loughborough, Leicestershire, LE11 3TU, UK; 5 Marwadi Education Foundation’s Group of Institutions, Rajkot Gujarat, India; 6 Department of Physics, Chemistry and biology (IFM), Linköping University, SE-581 83 Linköping, Sweden

## Abstract

Conductive polymers have been increasingly used as fuel cell catalyst support due to their electrical conductivity, large surface areas and stability. The incorporation of metal nanoparticles into a polymer matrix can effectively increase the specific surface area of these materials and hence improve the catalytic efficiency. In this work, a nanoparticle loaded conductive polymer nanocomposite was obtained by a one-step synthesis approach based on room temperature direct current plasma-liquid interaction. Gold nanoparticles were directly synthesized from HAuCl_4_ precursor in poly(3,4-ethylenedioxythiophene) polystyrene sulfonate (PEDOT:PSS). The resulting AuNPs/PEDOT:PSS nanocomposites were subsequently characterized under a practical alkaline direct ethanol fuel cell operation condition for its potential application as an electrocatalyst. Results show that AuNPs sizes within the PEDOT:PSS matrix are dependent on the plasma treatment time and precursor concentration, which in turn affect the nanocomposites electrical conductivity and their catalytic performance. Under certain synthesis conditions, unique nanoscale AuNPs/PEDOT:PSS core-shell structures could also be produced, indicating the interaction at the AuNPs/polymer interface. The enhanced catalytic activity shown by AuNPs/PEDOT:PSS has been attributed to the effective electron transfer and reactive species diffusion through the porous polymer network, as well as the synergistic interfacial interaction at the metal/polymer and metal/metal interfaces.

Over the last two decades, metal-polymer nanocomposites, in particular, metallic nanoparticles (NPs) embedded in a conducting polymer matrix, have attracted much attention for both academic and industrial investigations. Poly(3,4-ethylenedioxy thiophene) polystyrene sulfonate (PEDOT:PSS), a π-conjugated conducting polymer is of particular interest due to its high conductivity, high visible light transmissivity, excellent stability, and very good film-forming properties^1^. As a result, it is found in a wide range of applications including optoelectronic devices^2^,^3^, sensors^4^,^5^, thin-film transistors^6^, light-emitting diodes^7^, as well as electrode materials for organic solar cells^8^,^9^ or lithium batteries^10^.

The benefits of incorporating noble metal NPs into conducting polymers and PEDOT:PSS in particular, have been recognized over the last decade^11^,^12^,^13^,^14^. These nanocomposites are of great interest as they combine the properties of low-dimensional organic conductors with a high specific surface area^15^. Amongst the various metal NPs, gold nanoparticles (AuNP) are of particular interest and have been widely studied for chemical and biological sensing^16^, solar cells^17^,^18^, catalysis^19^, as well as for biomedical applications in imaging, cancer therapies and drug delivery^20^. These applications, to a great extent, depend on the broad material properties and the unique surface electronic structure of the AuNPs, which in part can be tuned by modifying the nanoparticle size. For instance, AuNPs embedded in PEDOT:PSS have been used in sensing applications due to the unique localized surface plasmon resonance (LSPR) properties of AuNPs^21^; the enhanced electrical conductivity of hybrid AuNPs/PEDOT:PSS, compared to pure PEDOT:PSS, makes it a superior material for thermoelectric applications^14^,^15^,^16^,^17^,^18^,^19^,^20^,^21^,^22^ and transparent film electrodes in biosensing^23^. Although much of the literature has been dedicated to the discussion of the AuNPs’ surface plasmons and their associated applications, the interface structure between AuNPs and polymer is much less understood. Nevertheless, the interface between the AuNPs and the polymer directly determines the relevant properties, especially the morphology and electrical conductivity of the resulting nanocomposite among other properties.

In recent years, metal NP/conductive polymer nanocomposites have attracted considerable interests in fuel cell catalyst applications. The highly porous surface (high specific area), good electrical conductivity and chemical stability of conductive polymers make them effective alternatives to the conventional carbon catalyst supports. The commonly reported conductive polymers include polyaniline, polypyrrole, polythiophene and their derivatives. The details of their properties, synthesis methods and characterization under fuel cell operation conditions have been described in ref. 24 and the references therein. Incorporation of highly dispersed metal NPs into conductive polymers maximizes the area for the oxidation and reduction reactions and makes low loading of catalyst possible for fuel cell applications^24^. Depending on the type of fuel cells and the electrodes (anode/cathode) used, a large range of metal NPs have been explored for their efficiency in fuel cell catalytic applications. In direct ethanol fuel cells for instance, Pt NPs and Pt-M (where M = Ru, Sn, W, Pd, Rh, Re, Mo, Ti, or Ce) alloyed NPs are the most extensively investigated^25^. Due to its lower cost and greater abundance compared to the traditionally used Pt catalyst, there is a surge of interest in deploying Pd in fuel cell applications. Pd is known to be more effective for ethanol oxidation than Pt under alkaline conditions due to its higher oxophilic nature which promotes the adsorption of -OH groups as well as its relatively inert nature with regard to C-C bond cleavage. Consequently, Pd is more stable and less susceptible to poisoning in the direct oxidation of ethanol to acetate^26^.

On the other hand, small Au based nanoparticles (<5 nm) have recently emerged as highly active catalysts for many important catalytic reactions^27^. A major factor for their catalytic activity is related to the size and composition-dependent properties of nanoparticles^28^. It has been found that AuNPs as the base catalyst in ethanol oxidation are inert in an acidic environment but active in a high pH (alkaline) medium. In addition, nanoscale gold has been shown to produce surface oxygenated species such as gold(III) oxide, adsorbed gold hydroxide or gold(III) hydroxide which are highly active for the removal of adsorbed CO (reduced poisoning), especially in alkaline media. Therefore AuNPs are considered as one of the alternative catalysts to Pt based NPs. It is known that controlled metal NP particle size distribution and solution dispersion are key determinants of catalytic efficiency. Typically, such requirements can be fulfilled through nanoparticle synthesis in bulk colloidal solutions in the presence of surfactants^29^. However the surfactant impedes the fuel gas access and electron transfer, which leads to seriously impaired electro-reactivity when the metal NPs are immobilized on conducting supports^30^.

In recent years, the role of Au in promoting the electro-oxidation reaction (EOR) of Pd nanocatalysts has been explored by several groups. Geraldes *et al*.^31^ prepared bimetallic PdAu electrocatalysts via electron beam irradiation and showed that a Pd:Au ratios of 90:10 gives the highest power density (44 mW cm^−2^ at 85 °C) compared to pure Pd (25 mW cm^−2^ at 85 °C). Dutta *et al*.^32^ found that ternary catalyst Pd:Ni:Au possessed a peak power density about three times that of the monometallic Pd catalyst. Zhu *et al*.^33^ synthesized a sub-monolayer Pd decorated AuNP electrocatalyst and found that a Pd:Au ratio of 1:4 gives the highest specific activity (peak current/Pd loading ~ 0.8 mA/μg) compared to pure Pd (peak current/Pd loading ~ 0.1 mA/μg) due to the electronic effect between the Au and Pd. Carbon-based supports have been used for dispersing the nanocatalysts in the above studies to achieve very high catalytic activity. However, conducting polymers can be an effective substitute to the carbon supports due to their lower ohmic drop across the electrode^34^.

Recently, several strategies have been reported on the *in situ* synthesis of AuNP/PEPOT:PSS nanocomposites, these include: electrochemical deposition of NPs onto electrodes previously coated with a conducting polymer; photo-electrochemical preparation; reduction of metal salts dissolved in a polymer matrix, mixing of nanoparticles into a polymer matrix; and simultaneous polymerization and nanoparticle formation^35^. These approaches normally require a range of additional chemicals (reducing agents, stabilizers etc.) and time consuming procedures (to add/remove surface chemistries and by-products). Other issues are related to hazardous/toxic chemicals used and potential contamination to the system. These factors add greater complexity to the process which could lead to reduced repeatability/reproducibility^36^, greater overall environmental and economic costs and less controllable NPs/nanocomposite properties^37^. Since the structures and properties of AuNP/PEDOT:PSS nanocomposites can be potentially influenced by multiple factors (e.g. synthesis method, valence state, inter-particle gap and the dielectric constant of the surrounding medium^38^,^39^), facile and more flexible nanocomposite synthesis routes which can lead to a better controlled material properties (hence functionalities) are highly desirable.

Previous work done by the authors has shown successful synthesis of surfactant-free AuNPs in aqueous solution using a room temperature atmospheric pressure microplasma technique where only the Au precursor was required^40^. In this work we present a one-step microplasma assisted fabrication process for stable AuNP/PEDOT:PSS nanocomposites, and provide the first report on the characterization of such nanocomposite towards applications in alkaline direct ethanol fuel cell (DEFC) catalyst. Normally, the inclusion of nanoparticles in such a polymer would disrupt its mechanical and electrical integrity. However the use of microplasma has proven effective in enabling the encapsulation of pre-synthesized nanoparticles (e.g. Ag, TiO_2_, BN) in PEDOT:PSS and other polymers, as demonstrated in our previous work^41^,^42^,^43^. We believe that the proposed technique opens a new avenue towards next generation hybrid nanocomposite fabrication and can be generalized for a wide range of NPs/polymer nanocomposite systems, where controlled materials physical and chemical properties are key in their functionalities.

## Experimental Details

### Precursor preparation

PEDOT:PSS (1.3 wt% dispersion in H_2_O) and HAuCl_4_·3H_2_O salt were purchased from Sigma-Aldrich. HAuCl_4_ aqueous solutions (50 mL) were prepared with different concentrations (2 μM, 20 μM and 200 μM) by dissolving the appropriate amount of HAuCl_4_·3H_2_O in distilled water. PEDOT:PSS (10 mL) were sonicated for 30 min and then filtered through a 0.2 μm pore size Millipore nylon filter (Sigma-Aldrich) to remove the precipitates. Then 80 μL of the filtered PEDOT:PSS solution was added to each one of the HAuCl_4_ aqueous solutions at different concentrations and magnetically stirred for one hour before plasma processing.

### Microplasma synthesis of AuNPs in PEDOT:PSS/aqueous solutions

**Figure 1:**
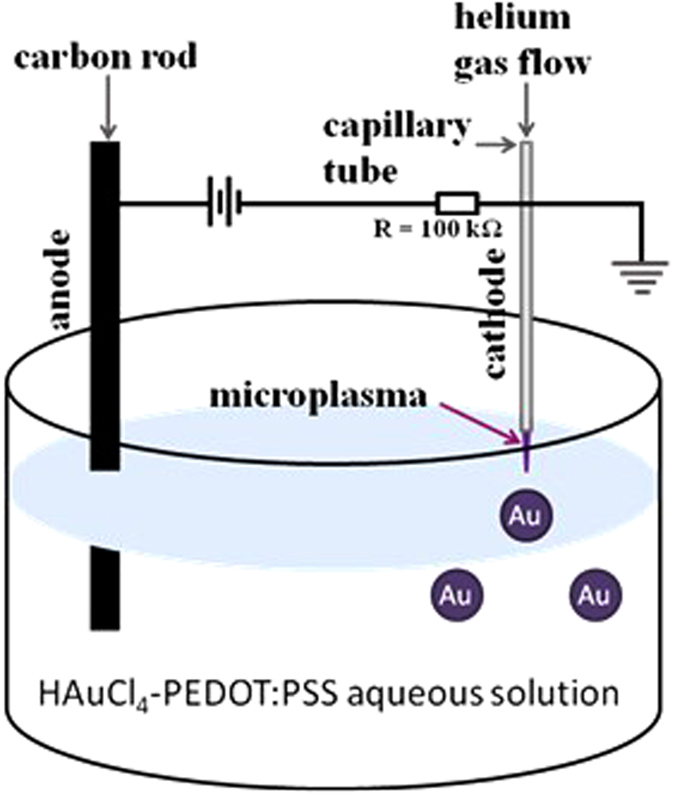
Schematic of the atmospheric pressure microplasma system.

Figure 1 shows the microplasma set-up used for the *in situ* synthesis of AuNPs. A room temperature atmospheric pressure direct-current (DC) plasma was initiated between a stainless steel capillary (0.25 mm inner diameter, 0.5 mm outer diameter) and the surface of the HAuCl_4_/PEDOT:PSS aqueous solution to synthesize the AuNPs. The gas flow through the stainless steel capillary (100% He) was held constant at 25 sccm. The detailed experimental set up can be also found elsewhere^42^,^43^,^44^. The distance between the capillary and the plasma-liquid interface was initially adjusted to 0.9 mm. A carbon rod was used as a counter electrode and kept at a distance of ~2 cm from the metal capillary, as shown in Fig. 1. Microplasma processing was carried out for different time intervals (i.e., 2 min, 5 min, 10 min and 20 min) on different samples at a constant current of 5 mA. The initial voltage was 2 kV and this was progressively reduced to 0.8 kV to maintain the current constant.

### Materials Characterization

Ultraviolet-visible (UV-Vis) spectroscopy (Perkin Elmer’s LAMBDA 35 UV-Vis spectrometer) has been used to study the absorption characteristics of the synthesized AuNPs in PEDOT:PSS aqueous solutions. Transmission electron microscopy (TEM) was performed on a JEOL JEM-2100F to analyze the size and size distribution of AuNPs.

The electrical conductivity of dried Au/PEDOT:PSS nanocomposites films (300 nm thick) were measured. 80 μL of aqueous PEDOT:PSS/AuNP samples was drop-casted on inter-digitated gold electrodes (IDEs) (Micrux ED-IDA5-Au) on glass and allowed to dry for 6 hours at room temperature before measurement. The IDEs consisted of 20 pairs of electrodes (electrode width was 10 μm, electrode thickness was 50 nm and the gap between electrode was 5 μm), covering an area of 5 mm diameter. DC current-voltage (IV) plots were obtained using a SemiLab DLS-83D DLTS semiconductor test system with an electrometer capability in the nA range.

Two of the AuNPs/PEDOT:PSS nanocomposite samples obtained from 2 μM HAuCl_4_ precursor, plasma treatment 5 min (Sample 1) and 200 μM HAuCl_4_ precursor, plasma treatment 5 min (Sample 2) were selected and their reactivity towards ethanol EOR was studied in alkaline medium under conditions typical of practical fuel cell operation. These composite samples were drop-casted on to a clean bulk Pd electrode and allowed to dry for 6 hours before further tests. Bulk Pd surface was employed as a bench mark catalyst (Sample 0) as it is the most active single bulk metal catalyst for ethanol EOR in alkaline media. Additionally, Pd is much cheaper and more abundant naturally than the conventionally used Pt based catalyst^45^. Our main aim is to investigate how the new catalysts can enhance the EOR activities of the bench mark material in alkaline DEFCs. Sample 0, Sample 1 and Sample 2 were characterized by electrochemical cycle voltammetry (CV) using a standard three-electrode cell with the chosen sample 0, 1 or 2 as the working electrode and Hg/HgO as the reference electrode and an Autolab electrochemical work station (Potentiostat, EcoChemie, Netherlands).

## Results and Discussion

**Figure 2:**
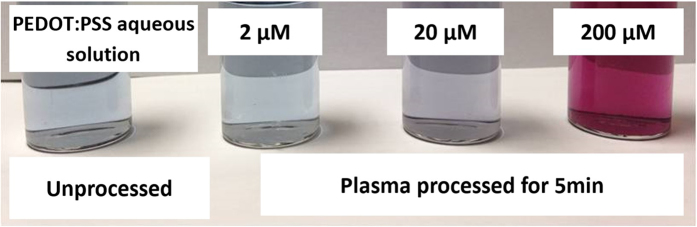
Colloidal gold nanoparticles (AuNPs) in poly(3,4-ethylenedioxythiophene).

**Figure 3:**
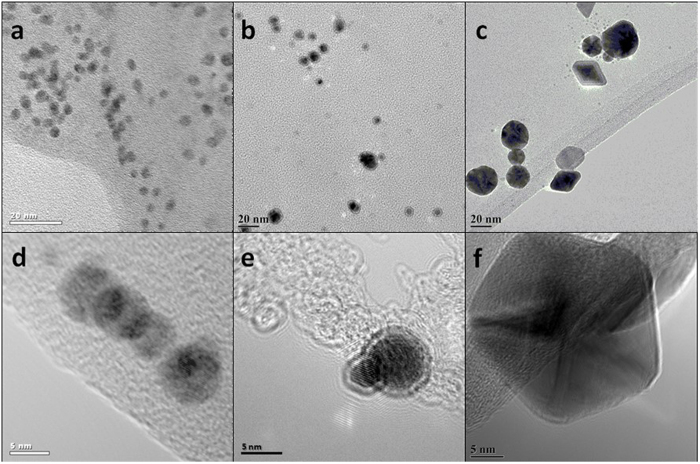
Low (top) and higher (bottom) magnification transmission electron microscope images of AuNP/PEDOT:PSS nanocomposites synthesized from different gold salt precursor concentrations (plasma treatment time 5 min).

Figure 2 shows the AuNP/PEDOT:PSS colloids obtained from various HAuCl_4_/PEDOT:PSS solutions after 5 min plasma treatment. The color change of the colloids indicates that the formation of AuNPs has taken place (confirmed also by TEM, see Fig. 3). Increasing the initial concentration of HAuCl_4_, the color becomes more intense with the sample containing 2 μM HAuCl_4_ showing negligible color change and the sample containing 200 μM HAuCl_4_ showing the most significant color change.

Corresponding TEM images are shown in Fig. 3 where both low and high magnification images confirm the formation of AuNPs at all three different concentrations. More details regarding statistical analysis of NPs size distributions can be found in Supporting Information S1. AuNPs produced with the lowest concentration (2 μM HAuCl_4_, Fig. 3a and d) are fairly uniform in size and being mostly spherical where the diameter of the NPs ranges within ~2–7 nm and with an average of 4.1 nm. For higher molar concentration of HAuCl_4_ (20 μM, Fig. 3b and e), the NPs are still spherical with a diameter within the range ~5–11 nm and an average of 7.5 nm (see Supporting Information S1). For 200 μM precursor concentration a large part of the AuNPs still remained spherical, however other shapes are emerging (hexagonal, pentagonal, triangular and rod shapes), see Fig. 3c and f. Although the diameter is more difficult to evaluate in this case, an average size of the spherical particles was estimated to be ~35 nm (see Supporting Information S1).

It is also found that for low gold salt precursor concentration, the resulting AuNPs appear to be uniformly dispersed/incorporated into a polymer matrix (Fig. 3d). With increasing precursor concentration, a distinct AuNP/polymer core-shell structure is produced (Fig. 3e). At even higher precursor concentration (200 μM, Fig. 3f), the core-shell structure becomes less visible. The reduction of the shell thickness could be a consequence of the increasing AuNPs to PEDOT:PSS concentration ratio, considering that the amount of polymer was constant (80 μL in 50 mL water). With increasing size and number of AuNPs, the average amount of polymer encapsulating each NP is reduced.

**Figure 4:**
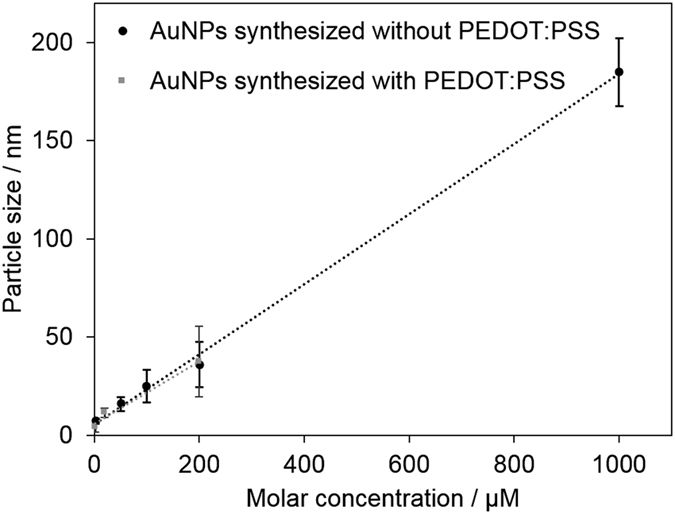
Average particle size vs. gold salt precursor concentration in aqueous solution with and without PEDOT:PSS (plasma processing time = 10 min). The data for AuNPs synthesized without PEDOT:PSS was taken from ref. 40.

In order to understand the effect of added polymer on the AuNPs formation, we compared AuNPs obtained from the present study with the ones obtained by plasma synthesis in polymer-free precursor solution (HAuCl_4_ in water only)^40^. Figure 4 shows the relationship between obtained AuNP size and the precursor concentration for the synthesis with/without PEDOT:PSS (with 10 min processing time). For both cases the average NP size is linearly dependent on the precursor concentration. The particle size follows the same linear relationship and it is clear therefore that the presence of the aqueous polymer has little impact on the growth rate for the same plasma conditions with mean nanoparticle diameters equal to those of the non-polymer case within the measured standard deviations.

The reaction pathway for the AuNPs formation in aqueous solution containing gold salt precursor has been proposed by Patel *et al*.^40^. At low concentration (e.g. 2 μM) reduction may be occurring predominantly in bulk solution, i.e. HAuCl_4_ may be reduced to yield Au^0^ atoms. The isotropic growth then leads to spherical particles. As the precursor concentration increases, partially reduced HAuCl_4_ may interact with the growing NPs leading to reduction on the surface of existing nucleated seeds; surface-assisted reduction will be at some extent dependent of the surface energy of the different crystal facets possibly leading to non-spherical shapes^40^. Since bare AuNPs obtained from aqueous solution (polymer-free) have negative surface charge (see Supporting Information, S2), it is reasonable to believe that the interaction between the AuNP surface and PEDOT:PSS molecules is via electrostatic interactions. In particular, the positively charged PEDOT component within the polymer might play an active role in binding with the AuNP, leading to the formation of the polymeric encapsulation. Such strong interfacial interaction between the nanofiller materials and the polymer matrix may also enhance the mechanical properties of the nanocomposites as well as improve their long term stability^46^,^47^.

It is known that the magnitude and the transport mechanism in PEDOT:PSS thin films are strongly anisotropic^48^. Generally, when PEDOT:PSS films are formed upon drying, they tend to organize in layers of PEDOT-rich oblate-spheroid or pancake-shaped particles (20–30 nm in diameter and 4–6 nm in height) separated by quasi-continuous nanometer-thick PSS lamellas^49^. The in-plane conductivity is described by three-dimensional variable range hopping between the PEDOT-rich particles separated by sub-nanometer PSS barriers, while the out-of-plane conductivity is described by nearest-neighbor hopping between more widely spaced molecular sites^49^.

**Figure 5:**
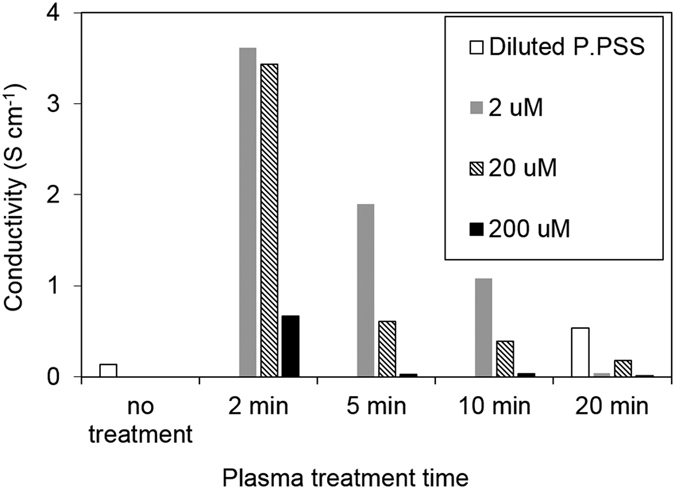
In-plane conductivity measurements on AuNP/PEDOT:PSS nanocomposite films obtained from colloid samples with different precursor concentrations drop casted/dried on IDEs.

Figure 5 shows the relationship between composite film electrical conductivity, gold salt precursor concentrations and plasma treatment conditions. Pure diluted PEDOT:PSS dry film was used as reference (conductivity ~0.16 S cm^−1^, the same order of magnitude with the as purchased PEDOT:PSS). Plasma treatment (20 min) of the pure diluted PEDOT:PSS caused slight increase in the dry film conductivity. The incorporation of AuNPs in the polymer has a strong impact on the resulting composite electrical conductivity. It can be seen that the lowest precursor concentration (2 μM) and shorter plasma treatment time (2 min) result in the highest conductivity enhancement. Increasing precursor concentration or plasma processing time decreases the film conductivity.

**Figure 6:**
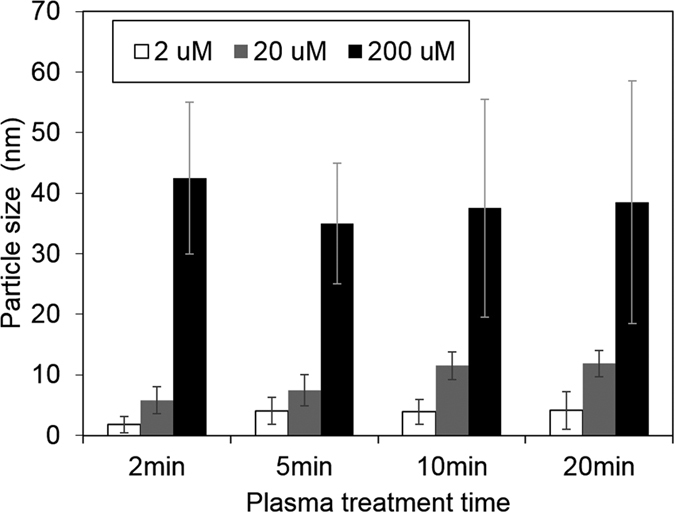
Mean AuNP particle size obtained from different plasma processing time and precursor concentrations.

The observed phenomena can be related to the AuNP sizes as well as the AuNP-polymer interactions. From the TEM images and the associated particle size analysis (see Fig. 6), it can be seen that increasing precursor concentration or plasma treatment time generally lead to increased AuNP sizes. When the AuNP particle size is small (e.g. <20–30 nm), the NP may interfere in the complexation of PEDOT with PSS forcing the poly(thiophene) moieties chains to assume a more extended conformation (which changes from a coil to linear or expanded-coil structure) that is associated with a more conductive quinoid^50^. In addition, when the NPs are small and less in quantity, they are incorporated in a continuous polymer film and well dispersed. The presence of AuNPs can promote the electron transfer through donor–acceptor interactions in the nanocomposites^51^.

As the AuNP size grows (e.g. greater than the size of the PEDOT particles), it may cause an increased inter-chain interaction resulting in conformational changes of the PEDOT chains. These larger AuNPs may have altered the domain arrangement within the PEDOT:PSS matrix. Also, the formation of core-shell structures may lead to a disrupted conductive polymer network, further deteriorating the overall composite film electrical conductivity.

**Figure 7:**
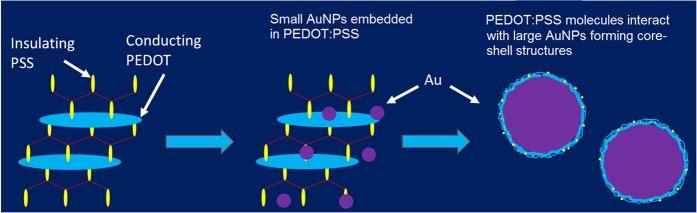
Schematic showing how AuNPs affect the PEDOT:PSS conformation and domain arrangement with increasing particle sizes.

Figure 7 gives schematic drawing showing the potential mechanisms contributing to the varying conductivity of nanocomposite films with different AuNP particle sizes.

Cyclic voltammetry (CV) is one of the commonly used techniques in electrochemistry and fuel cell electrocatalyst characterization. Under the alkaline type direct ethanol fuel cell operation conditions, the oxidation current peak in the forward CV potential scan can be used to evaluate the catalytic activity, as it is associated with the oxidation of ethanol and the adsorbed species produced from ethanol dissociation on the catalyst surface. The reverse scan may include the surface oxide (formed on the forward scan) reduction current overlapped with ethanol oxidation current^52^. Two AuNPs/PEDOT:PSS nanocomposites samples, namely Sample 1: AuNP/PEDOT:PSS (from 2 μM precursor, 5 min plasma treatment, average AuNP diameter 4.1 nm) and Sample 2: AuNP/PEDOT:PSS (from 200 μM precursor, 5 min plasma treatment, average AuNP diameter 35 nm), were selected. Both samples have been deposited on a clean bulk Pd electrode and allowed to dry completely for further tests. A bulk Pd electrode (Sample 0) was used as a bench mark catalyst for comparison. Nanoparticle catalytic activity is expected to be enhanced below a critical mean diameter of 5 nm^27^. We therefore compare a sub-5nm size distribution with a larger size distribution (e.g., 35 nm mean particle size). Full control of size distribution below 5 nm is under active investigation in a simpler aqueous chemistry system^53^ and advances can be expected to readily transfer to the polymer system.

**Figure 8:**
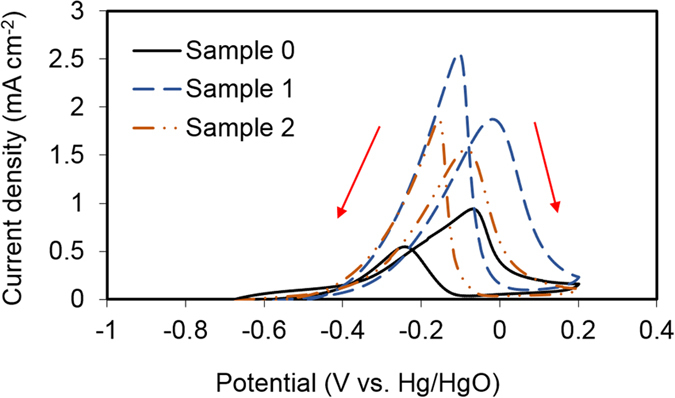
Cyclic voltammograms of bulk Pd (Sample 0), AuNP/PEDOT:PSS (Sample 1 with AuNP mean particle size 4.1 nm) and AuNP/PEDOT:PSS (Sample 2 with AuNP mean particle size 35 nm) deposit on bulk Pd in 0.1 M Ethanol +0.1 M NaOH solution at 30 °C. Scan rate: 50 mVs^−1^.

From Fig. 8 it can be seen that the onset potentials for EOR in the forward scan are similar for all samples. However, the forward current density peak of Samples 1 and 2 increases a lot more rapidly, and the peak current is about 2.0 and 1.5 times higher than that of the bulk Pd, respectively. In the reverse scan, the current density peak for Samples 1 and 2 shows even greater enhancement compared to the bulk Pd. It is obvious that the electrode surface composition greatly influences the behavior of ethanol oxidation. Also, it can be seen from Fig. 8 that the bulk Pd shows the lowest catalytic activity for EOR by giving the lowest peak current density under a similar peak potential. The catalytic activity of all samples tested for EOR are increased in the following order Sample 0 < Sample 1 < Sample 2 according to their peak current densities.

Pd is the most active single metal catalyst for ethanol electrooxidation in alkaline media. The detailed mechanism of ethanol oxidation on a Pd disc electrode has recently been investigated by Liang *et al*.^54^. From their CV characterization, it is suggested that the rate determining step is the removal of the adsorbed acyl by the adsorbed hydroxyl, while the dissociative adsorption of ethanol proceeds quickly. The mechanism for ethanol electro-oxidation is dependent on catalyst composition, leading to different reaction products, such as acetaldehyde and acetate, depending on the number of electrons transferred.

In our study, the enhanced electrocatalytic activity of AuNP on PEDOT:PSS support over bulk Pd has been attributed to the following reasons. Previous report has shown that AuNPs possess electrocatalytic activity (in the absence of Pd) for ethanol electro-oxidation in an alkaline medium^31^, where the ethanol oxidation has been attributed to the Au oxide formation or enhanced adsorption of OH^−^ species on the Au surface. In our study, since the AuNPs are in the proximity to the Pd bulk electrode, the electron transfer (resulting from the Au oxide formation) between Au and Pd is allowed in the conductive polymer network, this will in part contribute to the enhanced currents seen in the positive scans of Sample 1 and 2 in Fig. 8. Additionally, the greater amount of OH^−^ species attracted to the Au could also easily transport to the Pd surface through a diffusion process - the presence of porous PEDOT:PSS will not block the ion diffusion pathway. On the other hand, although the synthesized AuNPs are thought to be embedded in the PEDOT:PSS film during the sample preparation process, the physical contact between AuNPs and the Pd surface cannot be ruled out due to the porous nature of the PEDOT:PSS network. Au is the most electronegative metal, hence the electron-withdrawing effect from Au to the neighboring Pd cannot be neglected^55^. Such electronic interaction would lead to an interfacial synergy through the bi-functional mechanism, where the OH^−^ species adsorbed on the Au, adjacent to Pd would spill over to Pd and increase the concentration of OH_ads_ species on the Pd, facilitating overall ethanol electro-oxidation^31^. A better AuNP dispersion and smaller particle size would further increase the active area for ethanol adsorption, leading to enhanced EOR activity.

## Conclusions

In this work we have successfully demonstrated a one-step synthesis process for AuNPs-PEDOT:PSS nanocomposites and their application as alkaline direct ethanol fuel cell catalyst. Results show that by controlling the precursor/polymer concentration ratio and the plasma processing conditions, nanocomposites with wide range of structures/properties can be obtained. Smaller AuNP produced from low precursor concentration and shorter plasma processing time shows better composite film conductivity, as they are uniformly dispersed/incorporated in a continuous polymer film, enhancing the electron transfer efficiency. The decreased composite film conductivity with increasing precursor concentration and/or plasma treatment time maybe due to the encapsulation of AuNPs in polymer and formation of core-shell structure, disrupting continuous conductive polymer network, hence deteriorating the overall composite film electrical conductivity. When characterizing selected nanocomposite films as direct ethanol fuel cell electrocatalysts under alkaline conditions, use of AuNP/PEDOT:PSS on bulk Pd electrode has shown significant enhancement in EOR activity, with small AuNP size (<5 nm) giving better performance. These findings suggest that the AuNPs particle size/dispersion as well as the interactions at the AuNPs, conductive polymer and the Pd bulk electrode interfaces play an important role in the composite electrical and electrocatalytic properties. The enhanced catalytic activity shown by AuNPs/PEDOT:PSS is related to the effective transfer electrons and diffusion of reactive species through the porous polymer network, as well as the synergistic interfacial interaction between the metal/metal and metal/polymer interfaces. This study demonstrates that the AuNP/PEDOT:PSS nanocomposite synthesized through plasma-liquid interaction may serve as a promising candidate for a new class of Pt-free fuel cell catalysts. More importantly, this simple, rapid and environmentally friendly approach could be expanded to the synthesis of a much greater range of metal NPs/polymer nanocomposites with controlled structure/properties, providing enhanced functionality in various applications.

## Additional Information

**How to cite this article:** Zhang, R.-C. *et al*. Gold nanoparticle-polymer nanocomposites synthesized by room temperature atmospheric pressure plasma and their potential for fuel cell electrocatalytic application. *Sci. Rep.* **7**, 46682; doi: 10.1038/srep46682 (2017).

**Publisher's note:** Springer Nature remains neutral with regard to jurisdictional claims in published maps and institutional affiliations.

## Supplementary Material

Supplementary Information
