# Supplementary material for: Gold nanoparticle-polymer nanocomposites synthesized by room temperature atmospheric pressure plasma and their potential for fuel cell electrocatalytic application
Source: Sci Rep. 2017 Apr 24;7:46682. doi: 10.1038/srep46682 (PMC5402388; doi:10.1038/srep46682)
Supplement: Supplementary Information [file srep46682-s1.pdf]

SUPPORTING INFORMATION FOR

Gold nanoparticle-polymer nanocomposites synthesized by room temperature atmospheric pressure plasma and their potential for fuel cell electrocatalytic application

Ri-Chao Zhang[1,2,3], Dan Sun[2]*, Ruirui Zhang[4], Wen-Feng Lin[4], Manuel Macias-Montero[3], Jenish Patel[3], Sadegh Askari[5], Calum McDonald[3], Davide Mariotti[3], Paul Maguire[3]

1. School of Materials Science and Engineering, East China Jiaotong University, Nanchang 330013, China

2. School of Mechanical and Aerospace Engineering, Queen's University Belfast, UK BT9 5AH

3. Nanotechnology and Integrated Bioengineering Centre (NIBEC), Ulster University, Northern Ireland, UK, BT37 0QB

4. Department of Chemical Engineering, Loughborough University, Loughborough, Leicestershire, LE11 3 TU. UK

5. Department of Physics, Chemistry and biology (IFM), Linköping University, SE-581 83 Linköping, Sweden

**Corresponding authors: d.sun@qub.ac.uk*

S1. Particle size and size distribution analysis of gold nanoparticles

Figure S1 (a)-(c) show the TEM images of AuNPs obtained from the 2 µM, 20 µM and 200 µM $HAuCl_4$/PEDOT:PSS aqueous solutions, after 5 min plasma treatment. The corresponding particle size distribution (PSD; obtained from TEM analysis of 150 NPs) and Gaussian fit are presented in Figure S1 (d)-(f). Average diameters are obtained and reported in the paper.

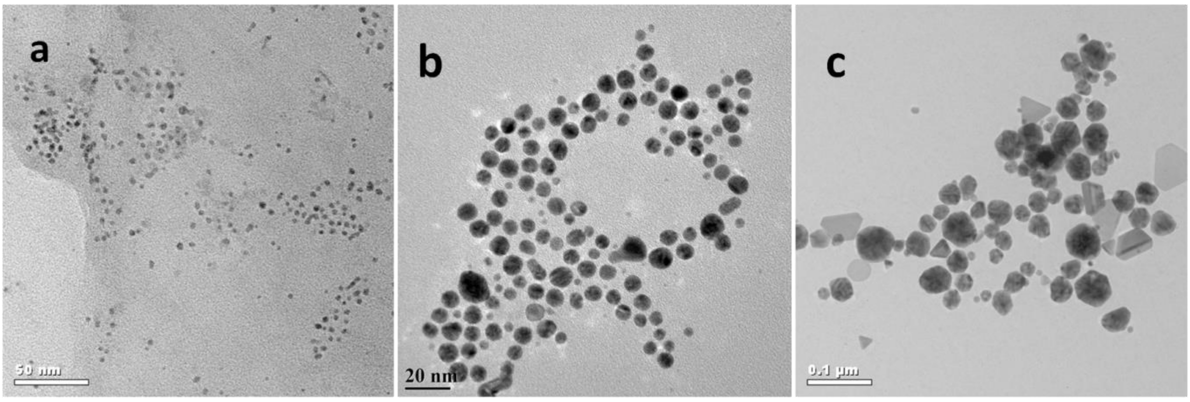

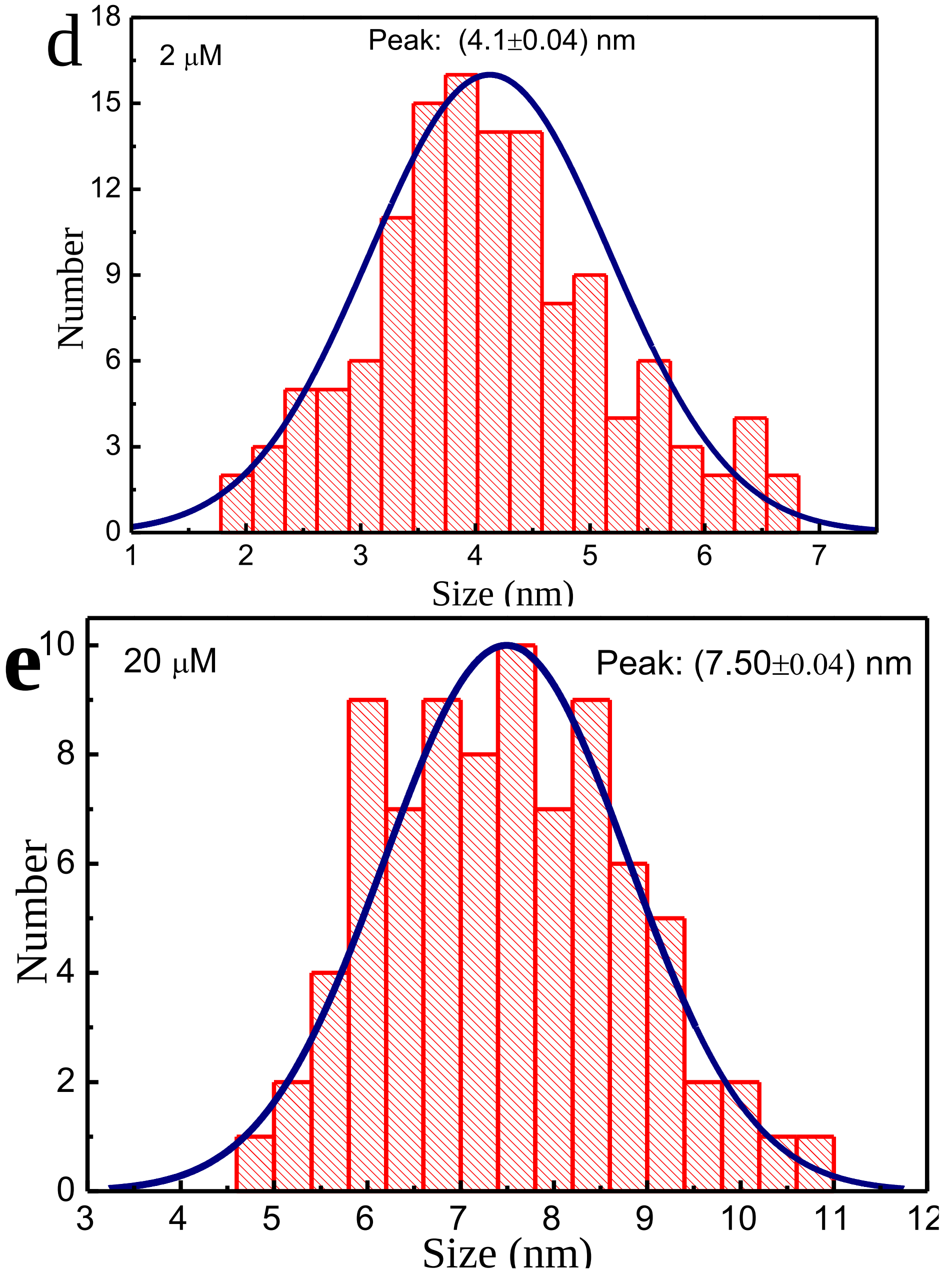
d
2 μM
Peak: (4.1±0.04) nm
Number
Size (nm)
e
20 μM
Peak: (7.50±0.04) nm
Number
Size (nm)

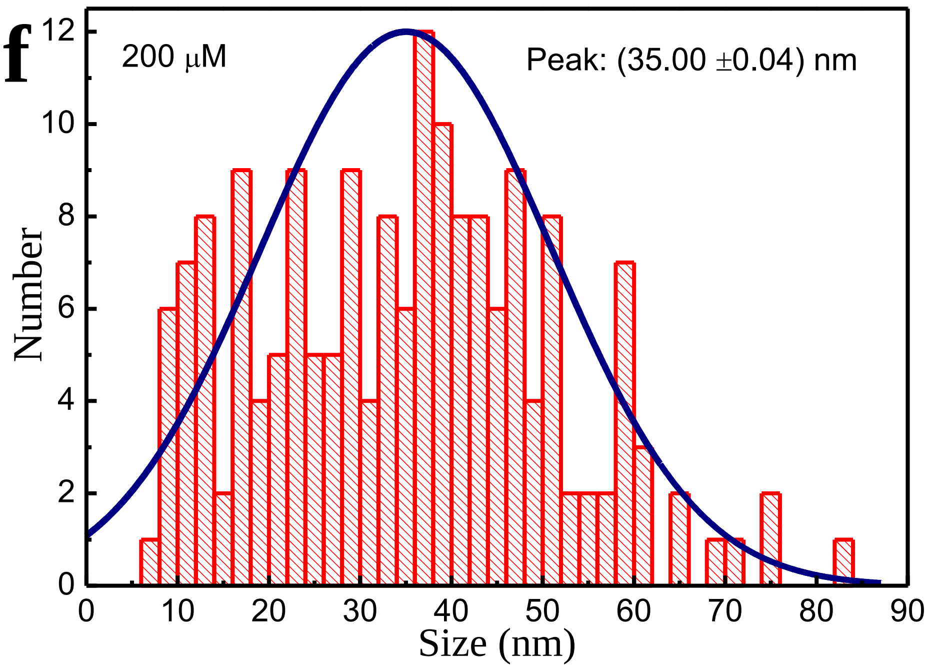

Figure S1. TEMs of AuNPs obtained from (a) 2 μM (b) 20 μM and (c) 200 μM gold salt solution after 5 min plasma processing and (d), (e), (f) their corresponding PSDs.

S2. Zeta potential measurements of gold nanoparticles

Figure S2 shows the Zeta potentials of AuNPs produced from 200 μM, 1 mM and 5 mM $HAuCl_4$/PEDOT:PSS aqueous solutions. The zeta potential of AuNPs increases from -41.8 to -29.5 as the concentration of gold salt solution increases.

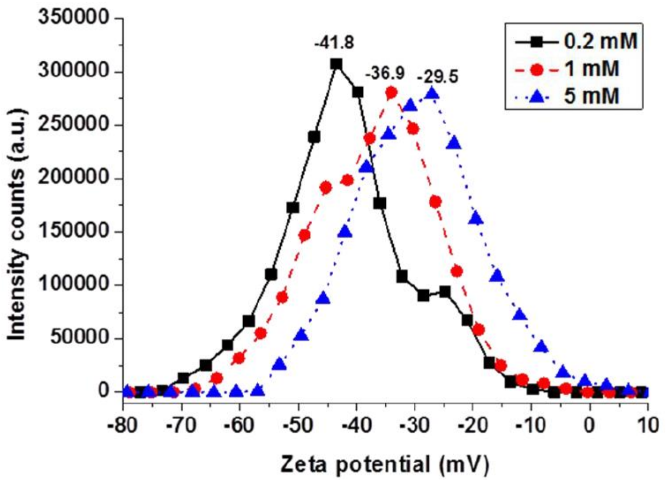

Figure S2. Zeta potential measurements of gold nanoparticles obtained from precursors with different concentrations
